# Risk-Aware Machine Learning Classifier for Skin Lesion Diagnosis

**DOI:** 10.3390/jcm8081241

**Published:** 2019-08-17

**Authors:** Aryan Mobiny, Aditi Singh, Hien Van Nguyen

**Affiliations:** Department of Electrical and Computer Engineering, University of Houston, Houston, TX 77004, USA

**Keywords:** Bayesian deep network, model uncertainty, Monte Carlo dropout, physician-friendly machine learning, skin lesion

## Abstract

Knowing when a machine learning system is not confident about its prediction is crucial in medical domains where safety is critical. Ideally, a machine learning algorithm should make a prediction only when it is highly certain about its competency, and refer the case to physicians otherwise. In this paper, we investigate how Bayesian deep learning can improve the performance of the machine–physician team in the skin lesion classification task. We used the publicly available HAM10000 dataset, which includes samples from seven common skin lesion categories: Melanoma (MEL), Melanocytic Nevi (NV), Basal Cell Carcinoma (BCC), Actinic Keratoses and Intraepithelial Carcinoma (AKIEC), Benign Keratosis (BKL), Dermatofibroma (DF), and Vascular (VASC) lesions. Our experimental results show that Bayesian deep networks can boost the diagnostic performance of the standard DenseNet-169 model from 81.35% to 83.59% without incurring additional parameters or heavy computation. More importantly, a hybrid physician–machine workflow reaches a classification accuracy of 90% while only referring 35% of the cases to physicians. The findings are expected to generalize to other medical diagnosis applications. We believe that the availability of risk-aware machine learning methods will enable a wider adoption of machine learning technology in clinical settings.

## 1. Introduction

In recent years, deep neural networks (DNNs) have gained tremendous attention and shown outstanding performances in many different computer vision tasks. These models are composed of stacks of processing layers to learn powerful representations from high-dimensional input data with multiple levels of abstraction [1]. Such models have quickly found their path to medical imaging and analysis applications such as lung nodule detection and classification in lung computed tomography (CT) scans [2,3], cancer detection in infrared spectroscopic images [4], and cerebral microbleeds detection in magnetic resonance (MR) images [5]. Deep networks have even matched or surpassed human-level performance in tasks such as diabetic retinopathy detection [6] and skin lesion classification [7]. Such systems can be employed to detect patients at risk from a prescreening examination, thus considerably decrease the physicians’ workload and diagnostic errors.

Computer-aided diagnosis (CAD) systems utilize sophisticated image processing and artificial intelligence techniques to assist doctors in the interpretation of medical images. Physicians use the output of CAD as a second opinion to improve the overall diagnosis performance by drawing the expert attention to abnormalities they overlooked, prompting them to re-evaluate the cases that might have been initially diagnosed incorrectly, and alleviating the inter-observer variability [8]. However, despite the recent successes reported in the literature, DNNs have not been extensively adopted in clinical settings thus far. One reason is that most of the existing studies focused on improving the *stand-alone* performance of CADs and comparing it against the human expert. However, the performance of CAD does not necessarily have to be comparable to or better than that by physicians, but needs to be complementary to that by physicians [8,9]. As a result, optimizing the quality of the interaction between physicians and CAD systems as a *team* is often overlooked.

Another reason for the slow uptake of the automated CAD systems is that DNN-based models tend to fail silently and have no risk-management mechanism [10,11]. In other words, they cannot inform doctors when they are not confident about their predictions. This raises the concern about the reliability of automated systems in real-life settings and situations with the possibility to become life-threatening to humans such as automated decision making or recommendation systems in the medical domain. An automated cancer detection system, for example, could encounter test examples which lie outside of its data distribution, thus make unreasonable suggestions and create harmful biases on physicians’ decisions. It is therefore desirable for DNNs to provide uncertainty measure in addition to the diagnostic decisions. Given this uncertainty measure, a physician could be informed at times when the system is essentially guessing at random [12,13].

**Figure 1:**
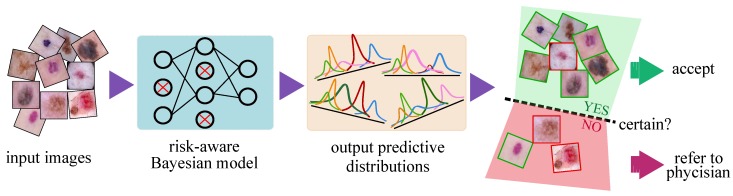
Processing pipeline of the proposed risk-aware Bayesian model. The Bayesian model outputs one predictive distribution per class (instead of the scalar outputs of the standard networks) whose mean and dispersion represents the network prediction and uncertainty, respectively. In the far right panel, the green (red) borders of the images illustrate the correct (incorrect) predictions of the automated model which is not always available as it requires manual annotation of samples by medical experts. The green (red) shaded areas, in contrast, depicts the regions where the model is certain (uncertain) about its prediction. Uncertainty is the natural output of the Bayesian model which serves as complementary information to refer the uncertain samples to experts and improve the overall prediction performance of the automated system.

This paper presents a lightweight, scalable CAD system which outputs an uncertainty estimate in the automated skin lesion diagnosis task (Figure 1). Based on this uncertainty, we investigate a hybrid physician–machine workflow where computers examine the majority of skin images and refer only difficult samples (i.e., predictions with lower confidence) to dermatologists for inspection. Displaying a confidence measure for each prediction facilitates more appropriate trust because physicians are less inclined to trust CAD diagnoses when they know that CAD does not have high confidence in it. Our model is simple to implement and incurs no additional complexity to the existing deep networks. The main contributions of this paper can be summarized as follows:

1. We propose a DNN-based CAD model that uses approximate Bayesian inference to output an uncertainty estimate along with its prediction in skin lesion classification. The proposed framework is general enough to support a wide variety of medical machine learning tasks and applications. Our results demonstrate the effectiveness of the confidence ratings in improving the diagnosis performance of the CAD–physician team and reducing the physician workload.
2. We formulate metrics to evaluate the uncertainty estimation performance of the Bayesian models. These metrics provide us with an informative tool to compare the quality of uncertainty estimations obtained from various models. Moreover, they provide hints for choosing an appropriate uncertainty threshold to reject samples and refer them to the physician for further inspection.
3. We provide in-depth analysis to show that the uncertainty-aware referral system via the Bayesian deep networks is effective for improving the team diagnosis accuracy on NV, BCC, AKIEC, BKL, and VASC lesion types.

The rest of this paper is organized as follows: Works related to uncertainty estimation and skin lesion classification are presented in Section 2. Section 3 explains the method used to approximate variational inference in Bayesian deep networks and generate uncertainty estimates for the skin lesion classification task. Data description and preparation is provided in Section 4. Experimental results are presented in Section 5 and discussed in Section 6. Section 7 concludes the paper with future research directions.

## 2. Related Work

### 2.1. Physician–CAD Interaction

Similar to the synergy between human experts in a multiple-reading setting, the combination of a physician (or a team of physicians) and CAD system creates a *diagnostic team* [9]. Many studies have shown the superior diagnostic performance of the CAD–physician team compared to the stand-alone physician performance [14,15,16]. However, there are studies that found CADs to have no benefit on experts’ diagnostic performance [17,18]. Observer studies show that the human experts’ level of *trust* in the CAD system is a key factor in improving team performance [9]. Doctors sometimes *under-trust*, CADs which consequently prevents them from utilizing their benefits. On the other hand, *over-trust* in automation leads to making diagnostic errors that would not have happened without CAD [18].

The way the output of CAD systems is presented to the human expert is a determining factor in building the appropriate level of trust and optimizing the team performance. Conventional CAD systems have a certain response criterion. The structure in the input image is considered normal or abnormal based on whether the extracted information meets the criterion or not. However, most CAD systems do not express their confidence to the predicted response, thus may unreasonably bias the physicians’ decision-making [9]. Various studies have found that doctors might put too much trust in CAD, thus accept many of the CAD predictions which decreases the overall diagnostic performance [18]. For example, Alberdi et al. [19] found that radiologists put too much trust in the CAD’s ability to detect abnormalities which eventually caused the radiologists assisted by CAD to have lower sensitivity than unaided radiologists.

Displaying a confidence measure along with the conventional CAD prediction can help physicians to adapt their trust according to the model confidence. Therefore, they can rely less on automation when it is less confident and vice versa. Several studies found that radiologists’ classification performance of lung nodules improved when they were assisted that provided a malignancy likelihood [20,21]. Similar observations were also reported for breast mass classification [22,23]. This shows that estimating the uncertainty of the automated model can be useful; however, it has been often overlooked when designing deep neural networks (DNN) models for health-care. Leibig et al. [12] found that uncertainty estimates can provide useful information in the task of diabetic retinopathy classification to reject the predictions when the network is uncertain. However, they did not evaluate the effect of uncertainty-informed referral in the CAD–physician team diagnostic performance. In this paper, we propose a DNN-based CAD system that outputs a precise confidence estimate along with its prediction for the skin lesion classification task. We then exploit the model uncertainty to evaluate the stand-alone performance of the CAD and compare it with that of the CAD–physician team to display its practical effectiveness.

### 2.2. Skin Lesion Diagnosis

Skin cancer, including both malignant melanoma and non-melanoma, is consistently ranked among the most widespread types of cancer in the past several years [24]. According to Bray et al. [25], skin cancer accounts for more than 7.5% of all new cancer cases and 1.3% of cancer-related deaths reported all around the world in 2018.

Computer-aided diagnosis (CAD) systems aim to improve the human experts’ performance in terms of diagnostic accuracy and speed by alleviating the inter-observer variability and addressing the limited availability of trained experts [26]. Performance of the conventional CAD systems relied on the intermediate image processing stages such as extraction of hand-crafted features [27,28]. In recent years, deep learning-based approaches have attracted considerable interest in the computer vision and machine learning community including the medical imaging domain [29,30,31]. Convolutional neural networks (CNN) can automatically extract the higher-level representations directly from raw input images [32]. These models have been adopted and used in an end-to-end fashion in the skin lesion diagnosis task [33,34]. Esteva et al. [7] achieved dermatologists-level diagnosis performance using an enormous dataset and a standard Inception-v3 [35] architecture. Later, Gessert et al. [36] employed an ensemble of CNNs and achieved the best performance on a much smaller dataset (HAM 10000 [37]) for the ISIC 2018 Skin Lesion Diagnosis challenge. Despite the recent successes in improving the stand-alone diagnostic performance of the DNN-based automated models, to the best of our knowledge, there has been no study on how machine and dermatologists work together as a team.

### 2.3. Uncertainty Estimation

In the context of machine learning, knowing when an autonomous model is uncertain, and thus likely to make an incorrect prediction is important; especially in medical diagnosis where safety is critical. Generally, there are two types of uncertainty in Bayesian modeling [38]. Model uncertainty, also known as Epistemic uncertainty, measures what the model does not know due to the lack of training data. This uncertainty captures our ignorance about which model generated our collected data, thus can be explained away given enough data [39]. Aleatoric uncertainty, however, captures noise (such as motion or sensor noise) inherent in the data and cannot be reduced by collecting more data [40]. Studies have used different methods such as test-time data augmentation [41] and directly learning a mapping from the input data [40] to reliably estimate the input-dependent predictive uncertainty of deep neural networks. In this paper, we mainly focus on the former type of uncertainty as medical data are often scarce, making the model uncertainty the dominant mode.

Traditionally, most of the studies on epistemic uncertainty estimation are inspired by Bayesian inference with Bayesian Neural Network (BNN) [42] as the classic example of such models. BNNs are the probabilistic variant of the traditional neural networks which attempt to produce a distribution over the output for any given input. However, such models are computationally expensive in practice due to a large number of parameters of neural networks, as well as the computationally intractable inference of the model posterior. Thus, much effort has been spent on developing scalable, approximate BNNs [43,44,45,46]. Variational inference is the most common approach used for approximating the model posterior using a simple variational distribution such as Gaussian [46]. The parameters of the distribution are then set in a way that it is as similar as possible to the true distribution. However, the use of the Gaussian distribution considerably increases the required number of parameters and makes it computationally expensive. Gal et al. [39] showed that Dropout [47], a regularization technique commonly used in DNNs, is equivalent to approximate variational inference in the deep Gaussian process [48]. This technique has been widely adopted and used in various medical applications where safety is crucial. For instance, it has been shown to reliably estimate the prediction uncertainty in drug discovery [49] and diabetic retinopathy [12]. In the segmentation setting, DeVries et al. [50] demonstrated that such uncertainty estimates can be exploited for predicting the segmentation quality of the skin lesions. A potential disadvantage of MC-Dropout method is that it often requires many forward-pass samplings, which makes it computationally expensive [51]. Another approach to estimate uncertainty is Multiplicative Normalizing Flows [52], which does not scale to very large convolutional networks. An alternative method is named Deep Ensembles [53], which trains several models and uses the variance of the output predictions as uncertainty estimates. However, this technique is quite resource-intensive as it requires storing several separate models and performing forward-passes through all of them to make the inference.

## 3. Materials and Methods

### 3.1. Uncertainty Estimation via Bayesian Neural Networks

Theoretically, training a standard neural network with L layer parameterized by the weights is equivalent to the maximum likelihood estimation (MLE) of the network parameters, resulting in a single set of best parameters. However, using such point estimates ignores any uncertainty that we may have in the proper weight values [54]. A Bayesian neural network is the probabilistic version of the artificial neural networks which places a prior distribution (often a Gaussian) over the network’s parameter [42] and outputs a probability distribution over model parameters that expresses our belief regarding how likely the different model parameter values are. Therefore, given a new test sample, a Bayesian neural network outputs a predictive posterior distribution over class membership probabilities by integrating over the posterior. Moreover, the dispersion of this predictive posterior reflects the reliability of the predictions, yielding the model’s uncertainty to its predictions. Such information is not available in a standard network as it only outputs a single value specifying such prediction.

In a Bayesian network, predicting the unknown label is equivalent to using an ensemble of an infinite number of neural networks with various configuration of the weights. This is computationally intractable for neural networks with any size. Therefore, so much effort has been put into approximating Bayesian deep networks to make them easier to train [55,56]. However, some of the approximation methods do not scale to very large convolutional networks and datasets.

### 3.2. MC-Dropout for Bayesian Neural Network Approximation

Recently, Gal et al. [39] showed that a feed-forward neural network (i.e., cascade of densely connected layers) with an arbitrary number of layers, arbitrary non-linearities, and dropout [47] applied to all the units is mathematically equivalent to approximate variational inference in the deep Gaussian Process model [48]. This idea is later extended to convolutional neural networks showing that dropout can be used at test time to impose a Bernoulli distribution over the weights of the convolutional neural network to obtain an approximation to the predictive posterior distribution without requiring any additional model parameters [51].

Dropout is a technique used in many deep models to avoid over-fitting in which the units of a neural network are randomly dropped (i.e., its activation is set to zero) with probability $p_{drop}$. This method, called Monte Carlo (MC) Dropout, suggests that dropout approximately integrates over the model’s weights, yielding an interpretation of the model uncertainty. In practice, implementing the MC-Dropout technique is straightforward as many modern neural network architectures already leverage dropout for regularization purposes. In a standard neural network with dropout, each unit is randomly dropped with probability $p_{drop}$ at training time. At test time, the dropout is switched off, meaning that the units are always present and the weights are multiplied by $\left(1-p_{drop}\right)$ [47].

In contrast to standard networks, when using the MC-Dropout method to obtain the model uncertainty for a given test sample $x^{*}$, the dropout mechanism is kept on and the prediction (i.e., forward pass) is performed multiple times. This process is commonly referred to as Monte Carlo sampling over the network parameters and results in an approximate predictive posterior distribution. The predictive mean ($\mu_{pred}$) over the Monte Carlo iterations is then used as the final prediction on the test sample:

(1) $$\mu_{pred}\approx \frac{1}{T}\sum_{t=1}^{T}p\left(y^{*}|x^{*},{\hat{w}}_{t}\right)$$

where *T* is the total number of MC sampling iterations and ${\hat{w}}_{t}$ is the shorthand notation for the weights of the network with dropout imposed to its units in the *t*th MC iteration (i.e., the *t*th forward pass). For each test sample $x^{*}$, the class with the largest predictive mean ($\mu_{pred}$) is selected as the output prediction. On the other hand, the dispersion of the distribution of predictions is a reasonable proxy for the model uncertainty. Similar to Gal et al. [10], we use predictive entropy (*H*) to quantify the model uncertainty as:

(2) $$H\left(y^{*}|x^{*},D\right)=-\sum_{c}p\left(y^{*}=c|x^{*},D\right)\;\log\;p\left(y^{*}=c|x^{*},D\right)$$

where *c* ranges over all classes. Generally, the range of the obtained uncertainty values is not fixed across different datasets, network architectures, number of MC sampling, etc. Therefore, we use the normalized entropy $H_{norm}\in \left[0,1\right]$ computed as $H_{norm}=\frac{H-H_{min}}{H_{max}-H_{min}}$ to report our results and facilitate the comparison across various sets and configurations.

### 3.3. Uncertainty Evaluation Metrics

The MC-Dropout technique provides a lightweight, scalable approach to estimate the uncertainty in deep neural networks. However, quantitative evaluation of the resulted uncertainty values is challenging. This is because unlike model predictions, there is no ground truth for the uncertainty estimates. Here, we propose metrics to evaluate the uncertainty estimation performance of the Bayesian frameworks. These metrics require only the ground truth label of the sample, the model prediction and the estimated uncertainty value, $H_{norm}$. Predictions can simply be divided to *correct* and *incorrect* by matching the ground truth and the model prediction. Likewise, we can apply a threshold $H_{T}\in \left[0,1\right]$ on the continuous uncertainty estimation values of $H_{norm}$ to split the predictions into *certain* ($H_{norm}<H_{T}$) and *uncertain* ($H_{norm}>H_{T}$) groups. Therefore, when making inference in the Bayesian setting, we generally face four scenarios which are incorrect-uncertain (iu), correct-uncertain (cu), correct-certain (cc), and incorrect-certain (ic) predictions.

In a Bayesian framework, if high model uncertainty is indicative of erroneous predictions, it can be leveraged to mimic the clinical workflow and select proper subsets of the samples with uncertain diagnoses for further testing by an expert. This procedure will eventually increase the prediction performance of the automated system, thus builds the experts’ trust in such systems. More specifically, we want the final automated system to:

**Proposition** **1.** *Predict correctly if it is certain about its prediction.*

**Proposition** **2.** *Be uncertain if the prediction is incorrect.*

It should be noted that the converse of the above two assumptions is not necessarily the case. In other words, if a model is making a correct prediction on a sample, it does not necessarily require to be certain on the same. A model might, for instance, correctly detect an object, but with relatively higher uncertainty. This can happen if the instance is rarely presented to the model in such pose or condition. The above propositions can be summarized as the following conditional probabilities:

(3) $$P_{H_{T}}\left(\mathrm{correct}|\mathrm{certain}\right)=\frac{P(\mathrm{correct},\mathrm{certain})}{P(\mathrm{certain})}=\frac{N_{cc}}{N_{cc}+N_{ic}}=R_{cc}\left(H_{T}\right)$$

(4) $$P_{H_{T}}\left(\mathrm{uncertain}|\mathrm{incorrect}\right)=\frac{P(\mathrm{uncertain},\mathrm{incorrect})}{P(\mathrm{incorrect})}=\frac{N_{iu}}{N_{iu}+N_{ic}}=R_{iu}\left(H_{T}\right)$$

where *N* and *R* represent the count and ratio for each combination. We can also measure the overall accuracy of the uncertainty estimation as the ratio of the desired cases (i.e., correct-certain and incorrect-uncertain) over all possible cases. We call this metric Uncertainty Accuracy (UA) and define it as:

(5) $$\mathrm{UA}\left(H_{T}\right)=\frac{N_{cc}+N_{iu}}{N_{cc}+N_{iu}+N_{cu}+N_{ic}}$$

Higher values of these metrics correspond to the model that performs better. Note that the above three metrics are defined as functions of $H_{T}$ as their value changes with uncertainty threshold, $H_{T}$. After computing the uncertainty estimation $H_{norm}$ for an input image, the prediction is certain if $H_{norm}<H_{T}$, and uncertain if $H_{norm}>H_{T}$ at each threshold $H_{T}$. Therefore, the value of the proposed metrics can be used to set a proper threshold and refer appropriate subsets for further inspection by medical experts.

### 3.4. Approximate Bayesian Network Building Strategy

We considered several popular, state-of-the-art deep neural network architectures in our experiments, including VGG-16 [57], ResNet-50 [58], and DenseNet-169 [59]. The fully Bayesian variant of these networks should be trained with dropout after every convolutional and fully-connected layer [39]. However, it has been shown that in practice it is too strong a regularizer that decelerates the training and eventually deteriorates the prediction performance of the model [51,60]. Therefore, we quantitatively analyzed the performance of several Bayesian variants with different configurations to find the ones with the best prediction performance in the classification task in hand. While there are an infinite number of possible configurations to examine, we investigated a handful of plausible ones to find the sub-optimal configurations by: (1) inserting/removing the dropout at different network locations; and (2) grid search on the dropout ratio with $p_{drop}\in \left[0.1,0.9\right]$ with step sizes of 0.1.

**Figure 2:**
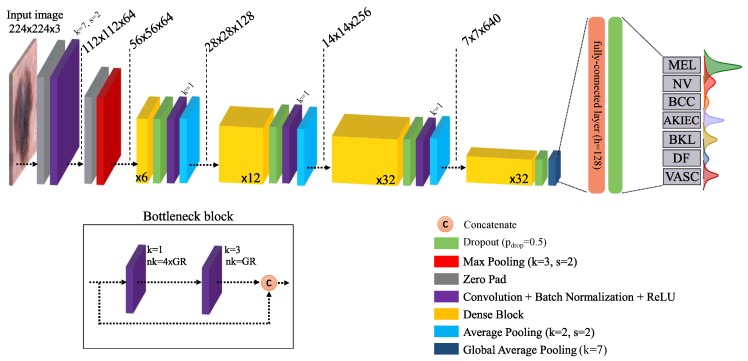
Schematic of the Bayesian DenseNet-169 architecture. The diagram shows the entire pipeline for the system which is trained end-to-end. Instead of a single scalar, Bayesian network outputs a predictive distribution per class whose mean and dispersion represents the network prediction and uncertainty, respectively. The number in the dense blocks corresponds to the number of bottleneck block within that dense block. *k*: kernel size, *s*: stride, nk: number of convolutional kernels, GR: growth rate [59].

The overall architecture of the final Bayesian DenseNet-169 model and its building blocks are presented in Figure 2 as the network which achieves the best performance in the lesion classification task (see Section 5 for detailed information on the results). Note that all Bayesian networks used in our study are actually *approximate* Bayesian models (as the exact Bayesian inference is computationally intractable for NNs). However, we drop the term “approximate” to avoid redundancy as is usually done in the literature. The standard DenseNet-169 network is composed of four Dense Blocks (DB) with a growth rate of 32 (see Table 1 in [59] for more detailed information). Each DB is followed by a convolution and average pooling pairs which together compress the information by reducing the spatial dimension and number of feature maps by half. The four DBs are composed of 6, 12, 32, and 32 bottleneck blocks, respectively. The bottleneck block was initially proposed by He et al. [58] and includes two convolution layers with filter sizes of 1 and 3, respectively. A global average pooling layer is used after the last DB, followed by fully-connected layers with, respectively, 128 and 7 (the total number of classes) units.

### 3.5. Training Procedure

As can be seen in Table 1, the strong class imbalance is a major challenge to be taken care of when dealing with this dataset. Therefore, we used loss balancing to compensate for the class imbalance. The utilized weighted cross-entropy loss function is defined as:

(6) $$L=-\sum_{c=1}^{C}w_{c}\;y_{c}\;\log\;p_{c}$$

where *c* is the class index, *C* is the total number of classes, $y_{c}$ is the ground truth label, and $p_{c}$ is the softmax-normalized model prediction. $w_{c}$ is the weight multiplied by the loss of class *c* and is defined as:

(7) $$w_{c}=\frac{N}{C\times N_{c}}$$

with *N* as the total number of training samples and $N_{c}$ the number of training samples in class *c*. Intuitively, this weighting strategy puts a stronger weight on the classes with fewer samples, thus puts more force on the network to predict them correctly.

We trained the network to minimize the weighted cross-entropy loss using ADAM [61] optimizer. We started the training with an initial learning rate of 0.001 and reduced it with a factor of 0.2 after each 10 epoch following a step-wise approach. Batch size was set to 128 and training was performed for the maximum of 100 epochs. We evaluated the validation accuracy after every epoch and saved the model with the best prediction accuracy on the validation set.

## 4. Data

### 4.1. Data Description

**Figure 3:**
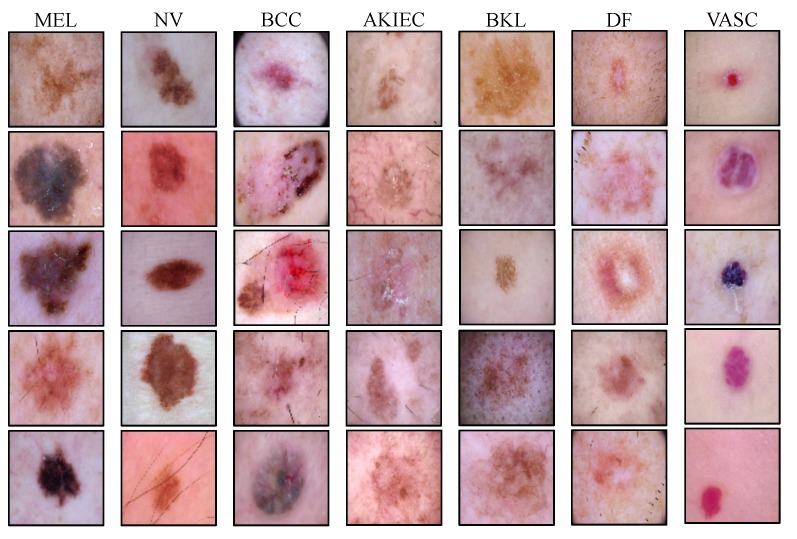
Illustrating examples from all of the pigmented skin lesion categories in the HAM dataset, including Melanoma (MEL), Melanocytic Nevi (NV), Basal Cell Carcinoma (BCC), Actinic Keratoses and Intraepithelial Carcinoma (AKIEC), Benign Keratosis (BKL), Dermatofibroma (DF), and Vascular lesions (VASC) classes.

We used the publicly available HAM10000 (Human Against Machine with 10,000 training images) [37] dataset for evaluating the accuracy of the automated diagnosis of pigmented skin lesions. This dataset contains 10,015 dermatoscopic images of the most important diagnostic categories in the realm of pigmented lesions collected from a diverse population and different modalities. Images are labeled by expert pathologists as one of the seven categories of Melanoma (MEL), Melanocytic Nevi (NV), Basal Cell Carcinoma (BCC), Actinic Keratoses and Intraepithelial Carcinoma (AKIEC), Benign Keratosis (BKL), Dermatofibroma (DF), and Vascular lesions (VASC). Example images of each of the seven lesion types and the number of available samples of each lesion type are shown in Figure 3 and Table 1.

### 4.2. Data Preparation

The original images are of size 600×450 pixels. We, however, resized all images to 224×224 pixels (using a bicubic interpolation over 4×4 pixel neighborhood), which is the common size used in ImageNet [62] challenge. This reduces the computational cost of the model and allows initializing the model parameters with those of the models pre-trained on ImageNet. The images were then standardized channel-wise using the mean and standard deviation values of the ImageNet dataset.

We initially split the whole dataset into training (80% of samples per class) and test (the remaining 20%) sets. We also made sure that images from the same lesion cannot occur in both training and test splits according to the information provided by the organizers [37]. At training time, we performed five-fold cross-validation where each fold includes an equal number of samples from each class. This means that the whole training set was randomly divided into training and validation sets five times and then a neural network was trained on each. Real-time data augmentation was also applied during training to mitigate over-fitting and improve the generalization of the model. Training images were randomly flipped along horizontal and vertical axes with a probability of 0.5, shifted along both axes, distorted with random changes in brightness and saturation, and randomly rotated around the center. At test time, each of the five trained models was evaluated on the disjoint test set and the final results are reported as the mean and standard deviation over the prediction accuracies.

## 5. Experimental Results

We start by describing the final Bayesian designs, evaluating their prediction accuracy and convergence performance. We also compare the performance of our proposed methods with that of the state-of-the-art (non-Bayesian) models used in other studies. Then, we analyze the obtained model uncertainty to see if it is useful for ranking the sample predictions, referring them for further inspection and correction, and improving the overall model performance. We finally shed light on the black box of the proposed Bayesian network to find the underlying causes of model uncertainty.

### 5.1. Bayesian Architecture Designs

We analyzed distinct probabilistic versions of the VGG-16, ResNet-50, and DenseNet-169 architectures according to the criteria explained in Section 3.4 to find the approximate Bayesian variants with the best prediction performance. Our experiments show that applying dropout to the initial convolution layers deteriorates the prediction performance of the networks regardless of the model architecture. Moreover, we observed that the ideal placement and ratio of the dropout layers depends on the model architecture. For VGG-16, the best prediction performance is achieved by placing the dropout layer with $p_{drop}=0.2$ before all max-pooling layers except the first one. We, however, achieve the best performance by dropouts applied after the residual and dense blocks of the ResNet-50 and DenseNet-169 with $p_{drop}=0.4$ and $p_{drop}=0.5$, respectively (see Appendix A.2 for more details on the final Bayesian architectures).

For each network, we also assessed the value of using pre-trained architectures. To do so, all network parameters were initialized by the weights of the model pre-trained on ImageNet [62], except the weights of the fully-connected layers, which were initialized randomly according to He et al. [63]. Similar to Gessert et al. [36], we found that fine-tuning the models pre-trained on ImageNet significantly outperforms the models trained from scratch.

### 5.2. Prediction Performance of the Bayesian Models

In this section, we investigate the inference performance of the proposed models and compare the prediction performance of our models with those achieved in other studies for the skin lesion classification task.

Table 2 summarizes the prediction accuracy of our implemented models (bottom), as well as that of the state-of-the-art models proposed in other studies (top). Among our models, Bayesian DenseNet-169 significantly outperforms the rest of the models. It also performs on par with or marginally better than the state-of-the-art models, except some of the models presented in [36], which exploit additional auxiliary processing stages to improve the performance (such as working with crops of the high-resolution images instead of down-sampling, conducting an extensive multi-crop evaluation, employing an ensemble of CNNs and a meta-learning step via training an auxiliary SVM classifier). However, as shown in the subsequent sections, our proposed Bayesian model is able to exceed their performance using uncertainty-aware referrals.

**Figure 4:**
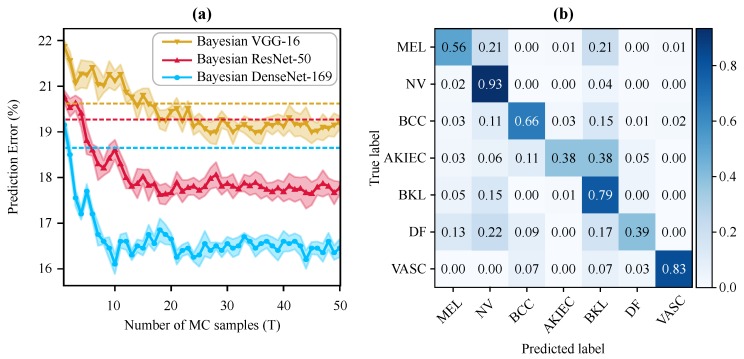
Test predictive performance of the Bayesian models in skin lesion classification. (**a**) Test prediction error of the Bayesian frameworks at different numbers of MC iterations (i.e., forward passes). The best result is achieved via Bayesian DenseNet-169 at 10 MC sampling. The shaded area around the curves shows one standard deviation. The dotted lines show the test error for the best standard (i.e., non-Bayesian) counterparts in the same color. (**b**) Normalized confusion matrix of the Bayesian DenseNet-169 on test data with 10 MC sampling.

Figure 4a illustrates the prediction performance of the Bayesian networks (as well as their non-Bayesian variant shown by dotted lines with the same color) for the different number of Monte Carlo simulations (*T*). Interestingly, the Bayesian DenseNet-169 outperforms the standard DenseNet-169 model after only two MC samples. Note that adding the Bayesian inference (i.e., MC-Dropout sampling) boosts the diagnostic performance of all three standard networks, with the Bayesian DenseNet-169 model performance improving by 2.24%. Moreover, Bayesian DenseNet-169 performs significantly more accurate than the other two Bayesian models at all *T*s. It also converges faster and achieves its lowest prediction error of 16.41% (classification accuracy of 83.59%) after only 10 samples while this number is 27 and 18 for Bayesian VGG-16 and ResNet-50, respectively. The normalized confusion matrix of the Bayesian DenseNet-169 at *T* = 10 is depicted in Figure 4b showing its per-class performance. We used this configuration as our best performing model for generating the results and analyses presented in the subsequent sections.

### 5.3. Uncertainty Estimation Performance of the Bayesian Models

In this section, we analyze the uncertainty estimation performance of the proposed Bayesian frameworks. We first describe how to generate and interpret the output predictive distributions (one per output class), and then use them to compute the model prediction score ($\mu_{pred}$) and uncertainty ($H_{norm}$). Finally, we perform an experiment to show that the model uncertainty measurements are often higher for incorrect predictions. This eventually implies that the model uncertainty could be exploited to mimic the clinical workflow and refer samples with uncertain diagnoses for further analysis to improve the overall diagnostic performance of the physician–machine team.

**Figure 5:**
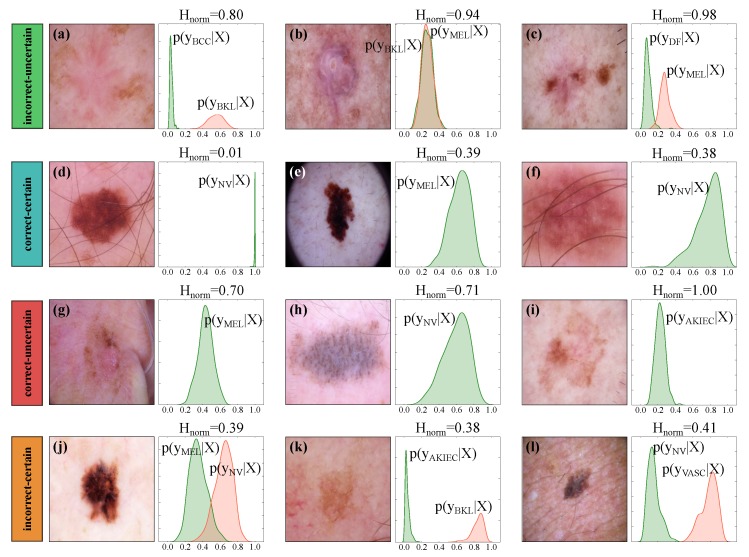
Illustrating sample output posterior distributions and the corresponding uncertainty estimates using the Bayesian DenseNet-169. The Bayesian inference outputs a posterior distribution (p(y|X)) per class where X represents the input image. We present the posterior distribution of the correct (in green) and incorrect (in red) classes. Predictions are grouped into incorrect–uncertain (**a**–**c**), correct–certain (**d**–**f**), correct–uncertain (**g**–**i**), and incorrect–certain (**j**–**l**) categories at threshold $H_{T}=0.5$. Note that Kernel density estimation with a Gaussian kernel is used to plot the output posterior distributions, for which the bandwidth was chosen according to Scott’s method [67].

In a standard neural network classifier, we obtain a single probability vector (of size equal to the number of classes) per input sample by applying a softmax normalization at the end of the network. In a MC-Dropout approximated Bayesian model, however, we obtain a predictive posterior distribution per output class by simply leaving the drop out on at test time. In other words, when the dropout is on, each forward pass results in a sample from the predictive posterior distributions. Figure 5 shows example input images and the corresponding predictive distributions generated by Bayesian DenseNet-169. While Bayesian model outputs seven distributions (one per output class), we only display the distributions associated with the true (in green) and predicted class (in red; only if the sample is misclassified). For each input sample, the class with the largest output distribution mean is selected as the output prediction and the dispersion of the output distributions (measured as in Equation (2)) depicts the model uncertainty. Intuitively, the wider the output posterior distributions of all classes, the less confident is the model in the prediction. For example, Figure 4d shows a correctly classified sample where the model is certain about its prediction ($H_{norm}=0.01$). In contrast, Figure 5i shows a correctly classified sample where the model is completely uncertain ($H_{norm}=1.00$).

Given the prediction scores ($\mu_{pred}$) and the normalized uncertainty estimates ($H_{norm}$), we can group the model predictions to incorrect–uncertain (iu), correct–uncertain (cu), correct–certain (cc) and incorrect–certain (ic) according to the criteria explained in Section 3.3. These groups are presented in Figure 5. As an example, a sample is grouped as “iu” if the prediction is wrong and the model is uncertain as well. While the ground truth label is not always available at test time, the estimated model uncertainty can serve as an informative hint to detect such predictions and refer them to medical experts. However, this statement is true only if high model uncertainty is indicative of incorrect predictions. This can be summarized as the two propositions presented in Section 3.3. Therefore, in a well-designed Bayesian model which satisfies these conditions, such uncertainty-aware predictions add complementary information to the output of the conventional deep networks and can be leveraged to increase the overall performance of the automated systems.

**Figure 6:**
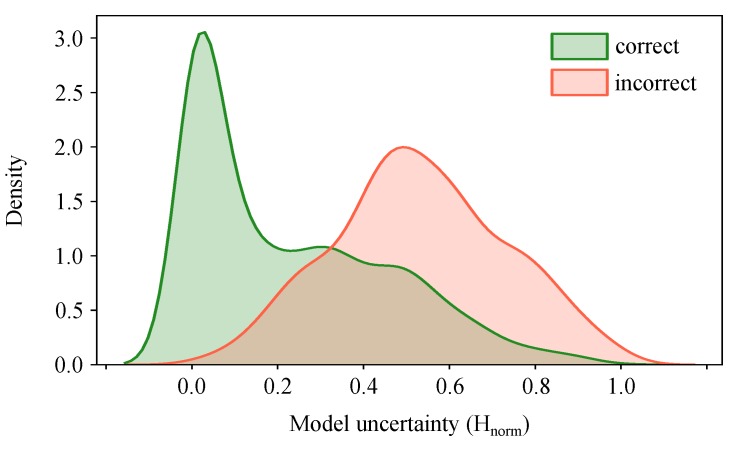
Distribution of normalized uncertainty values ($H_{norm}$) for all test samples grouped as correct and incorrect predictions. It shows that model uncertainty is higher for incorrect predictions. Therefore, it serves as complementary information to refer the uncertain samples to experts and improve the overall prediction performance of the automated system. Kernel density estimation with a Gaussian kernel is used to plot the output posterior distributions.

To check if our model satisfies the mentioned propositions, we plot the distribution of the uncertainty estimates for correct and incorrect predictions. Figure 6 shows that model uncertainty is indeed higher for incorrect predictions. This means that we can evaluate $H_{norm}$ at test time and leverage it to mimic the human clinical workflow by referring the uncertain predictions to the medical expert for further investigation.

**Figure 7:**
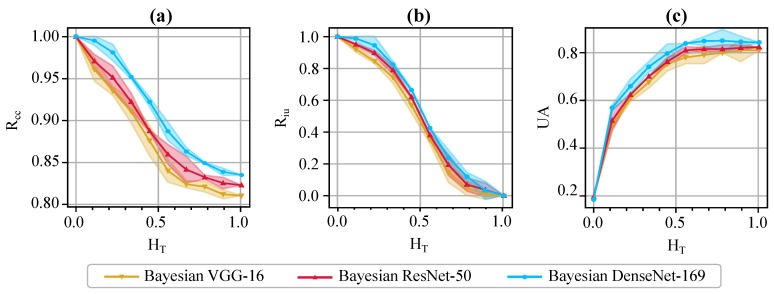
Quantitative evaluation of the uncertainty estimates via the proposed metrics. Illustrates the values of $R_{cc}$ (**a**), $R_{iu}$ (**b**), and UA (**c**) for varying thresholds of uncertainty ($H_{T}\in \left[0,1\right]$). The shaded area around each curve shows one standard deviation for the 5-fold cross-validation.

We also incorporate the evaluation metrics proposed in Section 3.3 (namely, $R_{cc}$, $R_{iu}$, and UA) to evaluate and compare the uncertainty estimation performance of various Bayesian models. We change the uncertainty threshold, $H_{T}$, in the range [0,1], and compute and plot the values of the evaluation metrics as in Figure 7. Note that, when $H_{T}=0$, all predictions are marked as uncertain. Hence, P(certain)=0 and the value of $R_{cc}$ is undefined. Therefore, we start the uncertainty threshold from 0.01 for the plot of $R_{cc}$. On the other hand, when $H_{T}=1$, all predictions become certain (i.e., P(uncertain)=0); thus, the values of both $R_{cc}$ and UA will be the same as the overall prediction accuracy of the model. Therefore, the proposed metrics serve as useful tools for the experts to decide about the proper value of the uncertainty threshold, and send useful, informative decision referrals to physicians. For example, $R_{iu}$ determines the fraction of incorrect predictions which fall in the uncertain category at various thresholds. It is over 80% and 90% at $H_{T}=0.3$ and $H_{T}=0.2$, respectively, for Bayesian DenseNet-169. On the other hand, $R_{cc}$ highlights the fraction of the certain predictions, which are indeed correct. The Bayesian DenseNet-169 model makes correct predictions 90% and 95% of the times when it is certain at thresholds of $H_{T}=0.5$ and $H_{T}=0.3$, respectively. However, the respective values are about 88% and 91% for Bayesian VGG-16, and 89% and 93% for Bayesian ResNet-50. This highlights the role of these metrics in comparing the quality of the uncertainty estimations for different network architectures or uncertainty estimation methods.

### 5.4. Uncertainty-Aware Skin Lesion Classification and Referral

We performed an experiment to examine whether a hybrid workflow, which combines Bayesian deep networks and dermatologists, would result in better accuracies compared to that of deep networks or dermatologists alone. Specifically, we first sorted all test samples according to their prediction uncertainty. Our system rejects a sample and refers it to dermatologists for further diagnosis when its uncertainty exceeds a certain threshold. If the uncertainty is lower than the threshold, the system accepts BNN’s prediction as the final outcome. The stand-alone prediction accuracy of the BNN is computed using only the accepted (i.e., non-referred) samples. To approximate the BNN–dermatologist team accuracy on the whole data, we need to know dermatologist’s diagnostic accuracy. Prior work shows that a dermatologist’s performance heavily depends on her level of experience in dermoscopy [68,69,70]. For example, experts with ≥5 years of experience perform significantly better than beginners with <2 years of experience [68,69,70]. Therefore, we computed the physician–machine team accuracy with different dermatologist’s accuracies varying from 60% to 80%, as reported in [7,70]. This eventually enabled us to understand the effect of dermatologist’s accuracy on the overall accuracy of the hybrid workflow.

**Figure 8:**
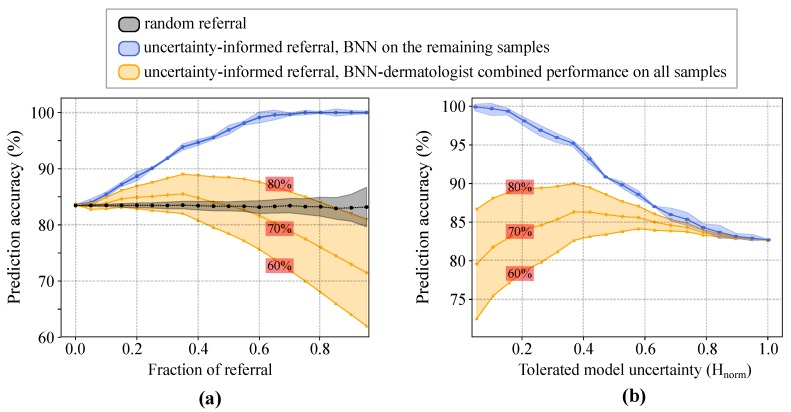
Enhanced prediction accuracy of physician–machine team via uncertainty-aware referral. (**a**) The classification accuracy as a function of the fraction of referral. The black curve shows the effect of rejecting the same number of samples randomly (i.e., with no use of uncertainty information). (**b**) The classification accuracy as a function of the tolerated amount of normalized model uncertainty. The shaded areas around the blue and black curves show one standard deviation for the five-fold cross-validation. For the BNN–dermatologist curves (shown in orange), the corresponding stand-alone performance of the dermatologist is shown in the red box on each curve.

As shown in Figure 8a, the stand-alone prediction accuracy of the BNN monotonically increases with the fraction of referred images. Note that only non-referred images are considered for computing machine’s accuracy. For example, if 20% of the data are referred to doctors, then we compute the accuracy using the remaining 80% of the dataset. We also compared the results with those of the random-referral; i.e., randomly selecting and rejecting the samples with no use of uncertainty information (Figure 8a, black curve). The experimental results show that, when only rejecting 5% of the samples for further inspection, the accuracy of the uncertainty-informed classifier is already significantly better than that of the random-referral counterpart. Moreover, the prediction accuracy goes up to 90% and 95% when referring 25% and 40% of the most uncertain samples for examination, respectively. In Figure 8b, the prediction accuracy of the model decreases monotonically with the increasing levels of tolerated model uncertainty. On the other hand, the BNN-dermatologist performance (shown in orange) depicts the impact of the dermatologist diagnostic performance on the overall team performance. For a beginner-level dermoscopy performance (i.e., 60% prediction accuracy), solely relying on BNNs will result in a more accurate overall diagnosis. However, for an experienced dermatologist (i.e., 80% accuracy), the team performance reaches almost 90% when rejecting either almost 35% of the most uncertain samples (see Figure 8a) or samples with $H_{norm}\geq 0.35$ (see Figure 8b).

### 5.5. Lesion-Specific Performance Analysis of Bayesian DenseNet-169

**Figure 9:**
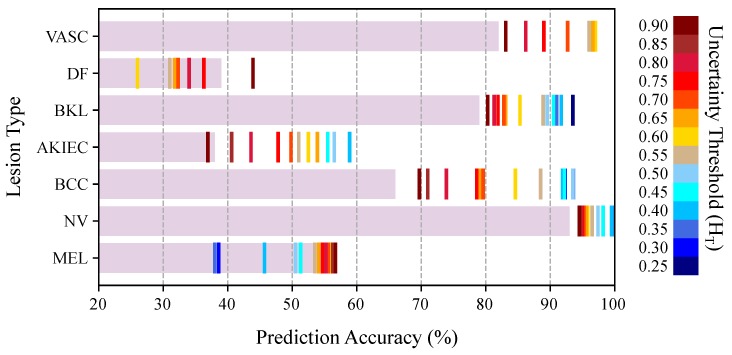
Prediction accuracy of the Bayesian DenseNet-169 model for various skin lesions as a function of the uncertainty threshold ($H_{T}$). The horizontal bars represent the performance of the Bayesian network on all test samples (no referral). The colored vertical lines depict the classification performance for each lesion at a specific uncertainty threshold.

**Figure 10:**
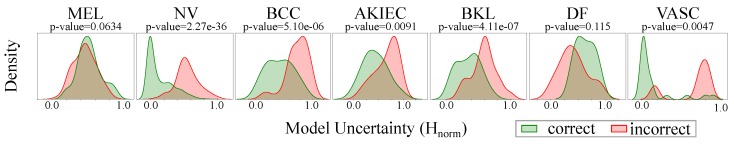
Distribution of normalized uncertainty values ($H_{norm}$) for each lesion type, grouped as correct and incorrect predictions. *p*-values are for the Kruskal–Wallis test between the correct and incorrect distributions for the null hypothesis that the population median of the two distributions are equal.

We analyzed the effect of uncertainty-based referrals on the diagnosis performance of different lesion types. Figure 9 depicts the stand-alone prediction performance of the BNN model on remaining samples at various uncertainty thresholds. As shown, the uncertainty-aware referrals help to improve the diagnostic performance of NV, BCC, AKIEC, BKL, and VASC lesions in a wide range of thresholds. However, the uncertainty-based referrals are not effective for DF and MEL categories. To find the reason, we plot the distribution of the uncertainty estimates for each lesion type in Figure 10. We also used Kruskal-Wallis [71] test to check if the distribution of the uncertainty values (correct vs. incorrect prediction) are significantly different for each lesion. The Kruskal–Wallis test was selected because it is non-parameteric and does not assume a particular distribution for the data. The null hypothesis is that the population medians of all of the groups are equal. The *p*-values are presented in Figure 10 for each lesion category. The resulted *p*-values show that we can not reject the null hypothesis for MEL and DF categories at 1% and 5% thresholds. This means that the Bayesian model is generally not able to output distinct (preferably higher) uncertainty values for the incorrect predictions of the DF and MEL lesion types, thus uncertainty-based referrals do not improve the model prediction for these categories.

**Figure 11:**
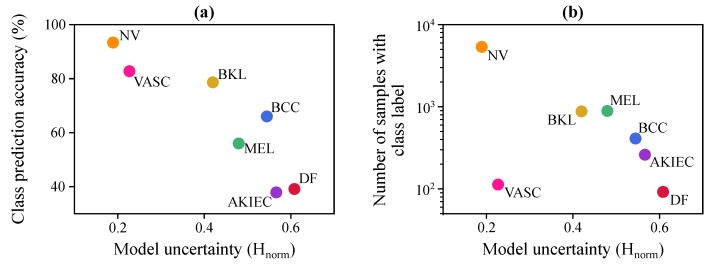
Analyzing the Bayesian DenseNet-169 model uncertainty in skin lesion classification. (**a**) Bayesian DenseNet prediction performance as a function of mean model uncertainty for each class. (**b**) Number of training samples in each class as a function of mean model uncertainty for each class.

To understand how the Bayesian model uncertainty changes with other factors, we plot the model uncertainty with respect to per-class model prediction accuracy and the number of training samples from each class in Figure 11. Uncertainty is computed as the mean uncertainty value for the samples of that class in the test set. This figure shows that there is an inverse relationship between class accuracy and model uncertainty (see Figure 11a), and a strong inverse relationship between the model uncertainty and the number of samples in each class of the training set, except for VASC (see Figure 11b).

## 6. Discussion

In this study, we showed that we can compute informative, interpretable uncertainty estimates for the skin lesion diagnosis task using the connection between the dropout operation and approximate Bayesian inference [39,51]. This method, commonly known as MC-Dropout [39], is scalable to large neural networks and input images and requires no additional labels or parameters. We observed that adding the MC-Dropout sampling immediately boosts the diagnostic performance of the popular standard neural networks by 1.39%, 1.92%, and 2.24% for VGG-16, ResNet-50, and DenseNet-169, respectively. More specifically, the Bayesian DenseNet-169 model obtains the largest prediction boost for the Basal Cell Carcinoma (+6.1%) and Benign Keratosis (+4.7%) lesions, but achieves no significant performance gain for the Melanoma and Vascular lesions.

Even though approximate Bayesian inference has the advantage of estimating the model uncertainty, it comes with the *potential* price of longer inference time. This is because we need to evaluate the network stochastically multiple times (shown as *T* in Equation (1)) and average the results to make the final prediction. Therefore, while the training time of the Bayesian framework stays the same, the test time is theoretically scaled by the number of averaged forward passes (*T*). This becomes more important in practice and in domains such as medical applications where the test-time efficiency is critical. However, this is not of major concern in real-world applications because deep networks are often implemented on distributed hardware [51]. Therefore, we can transfer the input image to a GPU, replicate it multiple times (i.e., *T* times) to form a mini-batch, feed the whole batch to the model at once and average the results. This eventually allows us to make the inference and obtain the uncertainty estimates in constant time. In our application, computing the predictive posterior (with T=10) for one image took less than 100 ms on a desktop machine with 128 GB of RAM memory and an NVidia GTX 1080 with 8 GB of video memory.

We observed (Figure 6) that model uncertainty is generally higher for incorrect predictions. Therefore, it is an effective measure of model confidence that can be used to inform physicians of times when the classifier is more likely to make mistakes. As a result, when referring the 20% and 25% of the most uncertain samples, the prediction accuracy of the automated model monotonically increases to 90% and 95%. This is in line with the findings in [72], which takes advantage of uncertainty-informed predictions to boost the model performance in an active learning setting. Uncertainty-based referrals have also been studied in the diabetic retinopathy detection task [12] and shown to be informative in detecting the models’ potential mistakes and improving the overall machine–physician performance. A toy 2D example in [12] revealed that the uncertainty-informed decision referral takes multiple separating hyperplanes into account, thus performs superior to the referrals made by the standard softmax outputs which take only one hyperplane.

We used the reports of the earlier studies to compute the physician–machine team accuracy for dermatologists with varying level of dermoscopy experience and prediction performance. A limitation of this approach is that the dermatologist–machine accuracy is computed under the assumption that the performance of dermatologists is independent of the referred images. This might not be the case in practice as referred images might be more difficult than normal images which result in lower dermatologist’s accuracy. Our experimental results demonstrate that for an experienced dermatologist with 80% diagnostic accuracy, it is best to reject 35% of the most uncertain CAD predictions so that the team performance reaches to almost 90% (Figure 8a). This means the hybrid workflow can save 65% of the physician’s time while increasing the diagnosis accuracy by 10% at the same time.

The analysis is then broken down to the lesion categories to investigate the effectiveness of uncertainty-based referrals for each lesion type. The results in Figure 9 and Figure 10 show that the model uncertainties are generally higher for incorrect predictions of five lesion types out of seven (namely, NV, BCC, AKIEC, BKL, and VASC). Therefore, as we refer more samples from the most uncertain model decisions, the model diagnostic accuracy percentage improves over the remaining samples of these lesion types (see Figure 9). However, the Bayesian model fails to output higher uncertainty values for the incorrect predictions of lesions of MEL and DF categories.

Analysis of the underlying causes of the model uncertainty reveals that the classes for which Bayesian DenseNet performs better, such as NV and VASC, are also the ones for which it is more confident. Conversely, for the more challenging classes, such as DF or AKIEC, Bayesian DenseNet shows a much higher model uncertainty. On the other hand, Figure 11b reveals a strong inverse relationship between the model uncertainty and the number of samples in each class of the training set, except for VASC. Thus, it can be inferred that the Bayesian model is often more confident about the samples that are more prevalent in the training set. Conversely, for the rarer classes, such as BCC and DF, Bayesian DenseNet is less confident. The only exception is the vascular lesions (VASC) class, in which the model makes quite confident predictions on average while the training set is relatively small. The reason is that, compared to other classes, the samples of this class have a relatively different appearance, which makes them easier to discriminate from samples of other classes. Therefore, this class becomes less ambiguous to the model, resulting in more confident model predictions.

This behavior of the uncertainty values estimated by the MC-Dropout method is consistent with the definition of model uncertainty where more training data are associated with less model uncertainty and vice versa. It eventually confirms that our approximate Bayesian model can effectively capture the uncertainty created by the lack of data in some classes. Similar observations were made by Kendall et al. [60] in estimating the model uncertainty in the semantic segmentation setting. In the road scene understanding task, the Bayesian model has been shown to be more confident about the more prevalent classes such as Sky or Road compared to the more rare classes such as traffic signs.

### Software and Code Availability

All models were implemented using the TensorFlow (version 1.13.1) and Keras (version 2.1.4) library [73]. Network training and prediction were performed using an NVIDIA GeForce GTX 1080 and with CUDA versions 9.0 and cuDNN 7.5. We will release the source code and trained models for public evaluation upon publication at https://github.com/hula-ai/skin_lesion_uncertainty_estimation.

## 7. Conclusions

In this paper, we present an approximate risk-aware deep Bayesian model, named Bayesian DenseNet-169, which outputs an estimate of the model uncertainty with no additional parameter or major change in the network’s architecture. Our classifier makes a prediction only when it is highly certain about its competency, and refers the case to physicians otherwise. Our experimental results in the skin lesion classification task show that the Bayesian model achieves high prediction diagnosis on par with the state-of-the-art models. We show that imposing approximate Bayesian inference increases the diagnostic performance of the standard DenseNet-169 model from 81.35% to 83.59%. Moreover, the prediction accuracy reaches nearly 90% and 95% on the remaining samples when exploiting the model uncertainty to refer, respectively, 25% and 40% of the most uncertain samples for further examination. This property enables a hybrid physician–machine workflow that saves human effort while maintaining high diagnostic accuracy. The proposed mechanism is general and applicable to any medical image classification task, involving microscopic, CT, MR, and ultrasound images. We expect that the availability of this technology will enable the wider adoption of machine learning technology in clinical settings. The future work will investigate the possibility of sending the uncertainty estimates to the network as feedback information to directly use it to modify and improve its prediction capability.

## Figures and Tables

**Table 1:** Class Distribution of HAM10000 dataset [37].

Lesion Type	MEL	NV	BCC	AKIEC	BKL	DF	VASC	Total
**Number of samples**	1113	6705	514	327	1099	115	142	10,015

**Table 2:** Quantitative comparison of the implemented models in skin lesion classification of HAM dataset. The ${}^{*}$ sign on some models shows that auxiliary processing stages and methods were exploited to improve the performance. Our models are shown in bold. $T^{*}$ represents the required number of Monte Carlo simulations to achieve the best performance in the Bayesian networks.

Method	% Prediction Accuracy (±std)
PNASNet [64]	76.00
ResNet-50 + gcForest [65]	80.04
VGG-16 + GoogLeNet Ensemble [66]	81.50
Densenet-121 with SVM ${}^{*}$ [36]	82.70
Densenet-169 ${}^{*}$ [36]	85.20
**VGG-16**	**79.63** (±0.25)
**ResNet-50**	**80.45** (±0.21)
**DenseNet-169**	**81.35** (±0.14)
**Bayesian VGG-16** (T${}^{*}$ = 27)	**81.02** (±0.22)
**Bayesian ResNet-50** (T${}^{*}$ = 18)	**82.37** (±0.14)
**Bayesian DenseNet-169** (T${}^{*}$ = 10)	**83.59** (±0.17)

**Figure A1:**
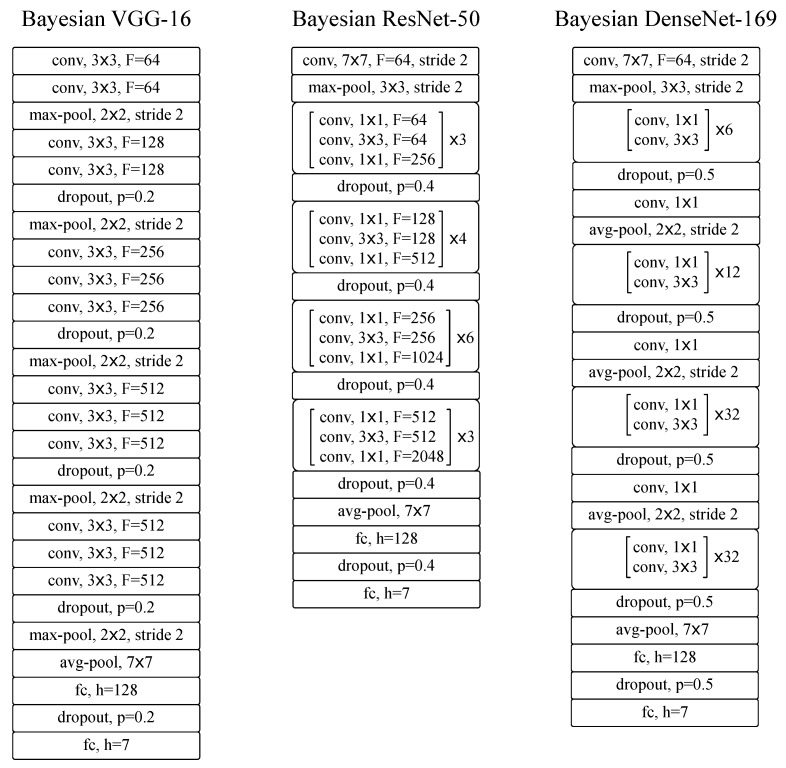
Designed Bayesian Network Architectures. In all convolution and pooling layers, stride is set to 1 unless otherwise mentioned. F represents the number of convolution filters and h is the number of units in the fully-connected layers.

## Abbreviations

The following abbreviations are used in this manuscript:

DNN	Deep Neural Network
BNN	Bayesian Neural Network
MC	Monte Carlo
iu	incorrect-uncertain
cc	correct-certain
ic	incorrect-certain
cu	correct-uncertain
UA	Uncertainty Accuracy

## Appendix A

### Appendix A.1. Dropout as Approximate Variational Inference in Bayesian Neural Networks

A Bayesian neural network is the probabilistic version of the artificial neural networks, which places a prior distribution (often a Gaussian) over the network’s parameter [42]. Given the entire training data D={X,y}, a Bayesian network produces a probability distribution over model parameters, p(w|X,y), that expresses our belief regarding how likely the different model parameter values are. Therefore, given a new test sample $x^{*}$, we can obtain the predictive posterior distribution over class membership probabilities by integrating over the posterior:

(A1) $$p\left(y^{*}|x^{*},D\right)=E_{p(w|D)}\left[p\left(y^{*}|x^{*},w\right)\right]=\int p\left(y^{*}|x^{*},w\right)p\left(w|D\right)\;dw$$

where $p(y^{*}|x^{*},D)$ is the predictive distribution that a test input data $x^{*}$ belongs to an unknown class $y^{*}$. According to Equation (A1), making a prediction about the unknown label is equivalent to using an ensemble of an infinite number of neural networks with various configuration of the weights. This is computationally intractable for neural networks with any size. Therefore, so much effort has been put into approximating Bayesian deep networks to make them easier to train [55,56]. Variational inference [46] is a technique commonly used to approximate the posterior on the weights p(w|D) with a variational distribution, $q_{\theta }\left(w\right)$, parameterized on θ, whose structure is easy to evaluate:

(A2) $$p\left(y^{*}|x^{*},D\right)\approx \int p\left(y^{*}|x^{*},w\right)q_{\theta }\left(w\right)\;dw$$

In the classification setting, Gal et al. [39] proved that minimizing the difference (i.e., KL-divergence) between the true posterior p(w|D) and the variational distribution $q_{\theta }\left(w\right)$ is equivalent to minimizing the conventional softmax loss in an L2-regularized neural network classifier with dropout [47] applied to its units. This method, called Monte Carlo (MC) Dropout, suggests that dropout approximately integrates over the model’s weights, yielding an interpretation of the model prediction and the associated uncertainty.

### Appendix A.2. Bayesian Model Architectures

Figure A1 represents the Bayesian architectures used in our experiments. A grid search is conducted on several network configurations (i.e., with various placements of dropout layers and drop ratio, *p*) to find the structures with the best prediction performance.
